# Supplementary material for: Deep Learning-Based Artificial Intelligence to Investigate Targeted Nanoparticles’ Uptake in TNBC Cells
Source: Int J Mol Sci. 2022 Dec 16;23(24):16070. doi: 10.3390/ijms232416070 (PMC9785476; doi:10.3390/ijms232416070)
Supplement: Supplementary file 1 [file ijms-23-16070-s001.zip › ijms-2019157-supplementary.pdf]

Supplementary Materials

Table S1. Accuracy and loss plots of the last fold of each model.

Model	Accuracy plots	Loss plots
Model 1	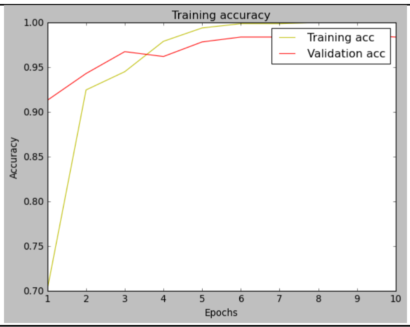	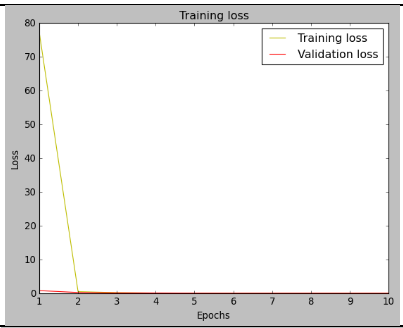
Model 2	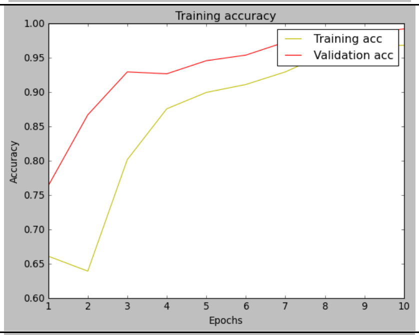	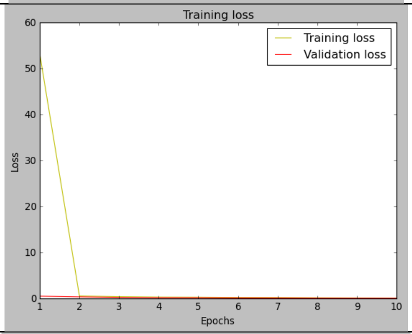
VGG16	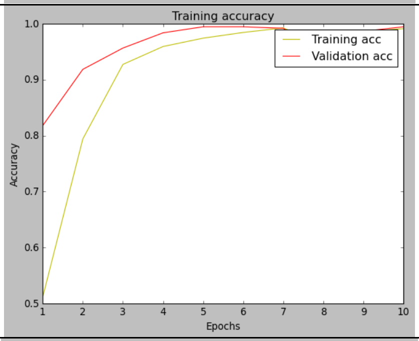	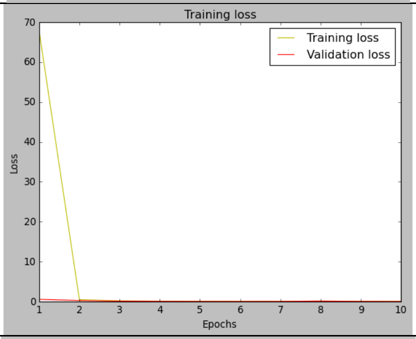
ResNet	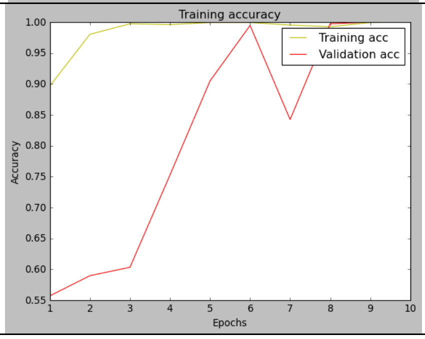	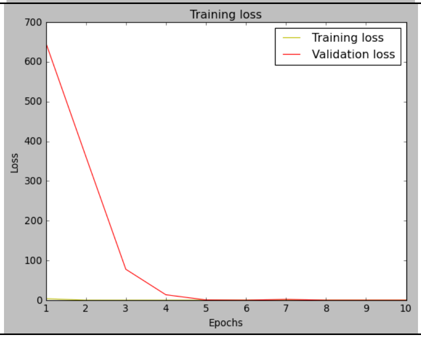

Inception V3	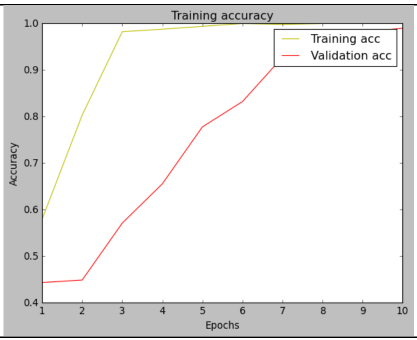	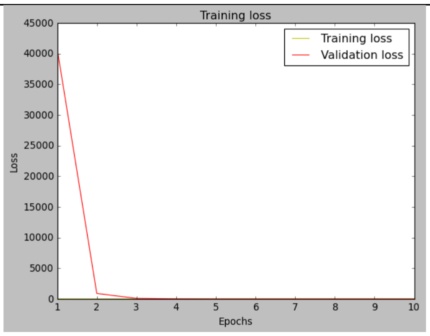
